# A Case of Gastric Emphysema: Incidental Findings or Serious Illness

**DOI:** 10.7759/cureus.11568

**Published:** 2020-11-19

**Authors:** Jonathan Vincent Reyes, Raju S Alluri, Ahmed AL-Khazraji, Tasur Seen, Aaron Walfish

**Affiliations:** 1 Internal Medicine, Icahn School of Medicine at Mount Sinai, Elmhurst Hospital Center, Elmhurst, USA; 2 Gastroenterology and Hepatology, Icahn School of Medicine at Mount Sinai, Elmhurst Hospital Center, Elmhurst, USA

**Keywords:** gastric emphysema, gastric pneumatosis, emphysematous gastritis

## Abstract

Gastric emphysema (GE) or gastric pneumatosis is a rare entity defined as air within the gastric wall. Etiologies include pneumothorax, instrumentation, infection, gastric wall ischemia, and mechanical injury. Several theories exist as to how the air disrupts the integrity of the gastric wall. These include a bacterial infection with Clostridium species and other gas-forming aerobic colonic bacilli, instrumentation with direct submucosal gastric wall injury, mechanical injury following increased intra-abdominal pressure, penetrating air through the mediastinum from increased intrapulmonary pressure or, gastric wall ischemia, which may be secondary to an underlying process. The diagnostic test of choice is CT of the abdomen. A hypodense linear fringe on the gastric wall is seen in GE, associated with gastric distention without thickening. In emphysematous gastritis, there is gastric wall thickening. There are no standardized guidelines for GE, but most cases have a good prognosis with a spontaneous resolution with conservation treatment. However, emphysematous gastritis management requires aggressive treatment due to the mortality rate of emphysematous gastritis approaching 60%. Patients are often considered for surgical intervention with total gastrectomy, given that active infection could delay or prevent healing. It is imperative to differentiate GE and emphysematous gastritis and understand the underlying pathogenesis as clinical outcomes are vastly different.

## Introduction

Gastric emphysema (GE) or gastric pneumatosis, defined as air within the gastric wall, is a very rare entity [1, 2]. This happens when gastric wall integrity is disrupted, leading to air passage into the gastric wall. Many etiologies lead to GE, such as pneumothorax, instrumentation, infection, gastric wall ischemia, and mechanical injury from excessive retching/vomiting [2]. It is important to clinically distinguish between gastric emphysema and emphysematous gastritis, given the high mortality associated with the latter. We report a case of a young female presenting with severe nausea and vomiting. An abdominal CT scan revealing air within the nondependent areas of the gastric wall. 

## Case presentation

A 24-year-old female with no past medical history presented to the emergency department (ED) with severe, continuous nausea and non-bloody, non-bilious (NBNB) vomiting for two days. She started having prior flu-like symptoms for five days with nonproductive cough, nasal congestion, fatigue, myalgia, and fever/chills. Subsequently, she was discharged from the ED with the diagnosis of flu. She later returned with new-onset NBNB vomiting, epigastric abdominal pain, and two days of watery diarrhea. She still had a residual cough and rhinorrhea. She endorsed an intolerance for solid food but tolerated clear liquids. She denied a history of recent travel and sick contacts. She denied ingestion of foods outside her ordinary diet, perished food, new medications, or caustic materials. However, she admitted to using marijuana four days before the onset of her symptoms and drinks alcohol daily of one-two glasses of wine. She did not use tobacco. She denied chest pain, shortness of breath, urinary symptoms, and prior similar presentation. On clinical examination, she was afebrile and hemodynamically stable. She did not have any pharyngeal erythema or lymphadenopathy. Cardiovascular and pulmonary exams were normal. The abdominal exam was remarkable for mild tenderness with deep palpation of the epigastric region.

**Figure 1:**
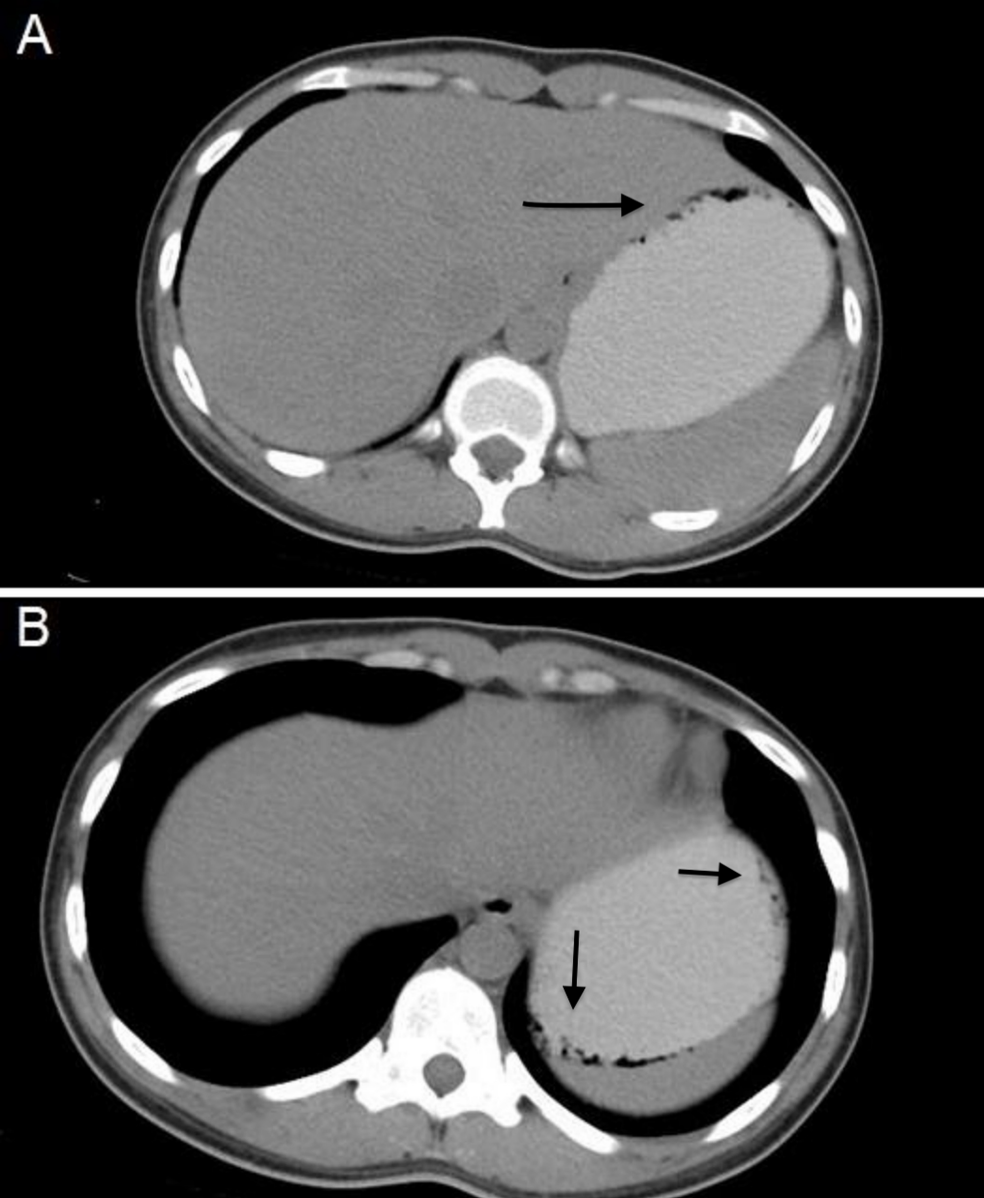
CT of the abdomen and pelvis with oral contrast A: CT of the abdomen and pelvis with oral contrast revealed bubbly gas within the gastric fundus and along the greater curvature concerning for gastric pneumatosis. B: CT of the abdomen and pelvis with oral contrast reported submucosal gas in the nondependent portion with trace submucosal gas identified in the dependent portion of the greater gastric curvature.

Laboratory data were unremarkable, including complete blood count, basic metabolic panel, liver enzymes, and lipase. The serum pregnancy test was negative. A urine drug screen was positive for opiates and marijuana. Abdominal/pelvic CT scan with oral and intravenous (IV) contrast demonstrated submucosal gas in the nondependent portion within the gastric fundus and concerning for gastric pneumatosis (Figure 1).

The patient was placed on bowel rest, and her pain was controlled with IV morphine. In addition to IV fluids, she was given empiric broad-spectrum antibiotic coverage with ampicillin-sulbactam. Upper endoscopy (EGD) was not recommended at the time, given that the risks of perforation would outweigh the benefit in the setting of gastric emphysema. Peripheral parenteral nutrition (PPN) was initiated on day three of hospitalization. The patient’s symptoms markedly improved, and she was discharged with a follow-up with her primary care physician after one week of discharge, which revealed the resolution of symptoms.

## Discussion

The radiologic finding of air within the gastric wall is seen in serious conditions such as emphysematous gastritis and gastric emphysema or gastric pneumatosis, where the latter has a more benign clinical course. There are no standardized treatments or guidelines for the management of gastric emphysema, given that the current evidence is limited to a few case reports and case series [2, 3]. There are several prevailing theories as to how the air disrupts the integrity of the gastric wall. First, a bacterial infection is seen with Clostridium species, as well as other gas-forming aerobic colonic bacilli, including Escherichia coli, Streptococcus, Bacillus subtili, and Bacillus proteus. The intraluminal gas produced by these species is a primary component of emphysematous gastritis [3, 4]. Second, instrumentation with direct submucosal gastric wall injury with enteral tube feeding or endoscopic procedures can results in GE [4, 5]. Third, mechanical injury following increased intra-abdominal pressure can force air into the gastric wall, resulting in excessive vomiting, such as in our patient. Also, increased abdominal pressure can occur rarely following non-invasive positive pressure. Fourth, penetrating air through the mediastinum into the gastric wall from increased intrapulmonary pressure in the pathological fibrotic lung (e.g., chronic obstructive lung disease (COPD)/asthma) and pneumothorax can eventually lead to air passage into the gastric wall [5]. Fifth, gastric wall ischemia secondary to systemic hypotension, vasculitis, and disseminated intravascular coagulation (DIC) can also contribute to GE's development [6].

The clinical presentation of gastric emphysema ranges from mild symptoms such as nausea, vomiting, and mild abdominal pain to severe with hematemesis, melena, and acute abdomen. Most cases follow a subacute to the acute course. Physical examination rarely supports the diagnosis. The diagnostic test of choice is CT of the abdomen as it can detect a minimal amount of air within the gastrointestinal tract and evaluate the abdominal cavity as well. A hypodense linear or curved fringe on the gastric wall is seen in GE, associated with gastric distention without thickening of the gastric wall. This contrasts with the radiologic findings of emphysematous gastritis, including gastric wall thickening or air in another segment of the bowel or biliary tract. There are no standard guidelines in terms of management of GE, and most cases have been treated conservatively. On the other hand, the management of emphysematous gastritis requires aggressive treatment given the high mortality of up to 60% of reported cases; it is still debated whether patients should undergo surgical intervention with total gastrectomy, given that active infection could delay or prevent healing [4, 5]. GE's prognosis is good with spontaneous resolution without specific treatment, in contrast to the high mortality rate of emphysematous gastritis [4]. 

## Conclusions

It is imperative to differentiate gastric emphysema and emphysematous gastritis as clinical outcomes are vastly different. As these two diseases may appear similar on imaging modalities, it is important for clinicians to understand the underlying pathogenesis of these two unrelated diseases to make a timely diagnosis. Gastric emphysema is typically self-limiting, while emphysematous gastritis requires aggressive therapy with intravenous antibiotics, intravenous fluids, and close monitoring as it has a devastating course.

## References

[1] Ghneim A, Meegada S (2019). Gastric emphysema induced by severe vomiting. Cureus.

[2] Inayat F, Zafar F, Zaman MA, Hussain Q (2018). Gastric emphysema secondary to severe vomiting: a comparative review of 14 cases. BMJ Case Rep.

[3] Murnan S, Miller J, Kuhn A (2019). Gastric emphysema: a cannot-miss emergency medicine diagnosis. Pediatr Emerg Care.

[4] Misro A, Sheth H (2014). Diagnostic dilemma of gastric intramural air. Ann R Coll Surg Engl.

[5] López-Medina G, Díaz de León RC, Heredia-Salazar AC, Hernández-Salcedo DR (2014). Gastric emphysema a spectrum of pneumatosis intestinalis: a case report and literature review. Case Rep Gastrointest Med.

[6] Tang SJ, Daram SR, Wu R, Bhaijee F (2014). Pathogenesis, diagnosis, and management of gastric ischemia. Clin Gastroenterol Hepatol.

